# GFOD1 and peejar are promising markers for clear-cell renal cell carcinoma disease progression

**DOI:** 10.18632/oncotarget.9379

**Published:** 2016-05-15

**Authors:** Xiao-rong Wu, Yong-hui Chen, Wei Chen, Wen Kong, Ji-wei Huang, Jin Zhang, Wei Xue, Dong-ming Liu, Yi-ran Huang

**Affiliations:** ^1^ Department of Urology, Ren Ji Hospital, School of Medicine, Shanghai Jiao Tong University, Shanghai, 200127, China

**Keywords:** gene expression profiling, long non-coding RNA, clear cell renal cell carcinoma

## Abstract

Clear cell renal cell carcinoma (ccRCC) is a common genitourinary malignancy. The molecular pathogenesis of ccRCC remains unclear and biomarkers for daily practice were still limited. We performed an integrative analysis of two public ccRCC microarray datasets, E-GEOD-22541 and E-MTAB-1050, The candidate differential expression genes (DEG) were then confirmed in the E-GEOD-53757 dataset. In addition, an independent cohort of 50 ccRCC and 36 non-tumor kidney tissues were analyzed to examine the selected DGEs by qRT-PCR. We identified and validated two DEGs, namely GFOD1 and peejar, which were significantly up-regulated in ccRCC compared with normal renal tissues (*p* < 0.001). Moreover, the expression of these two genes are related to histological grade and stage and decrease of their expression correlated with disease progression (*p* < 0.05). Furthermore, we found the expression of peejar was positively correlated with the expression of GFOD1 in ccRCC tissue, with Pearson correlation coefficiency reaching 0.939 (*p* < 0.001). GFOD1 and peejar were novel genes correlated with ccRCC disease progression and patients' poor prognosis.

## INTRODUCTION

Renal cell carcinoma (RCC) is one of the most common genitourinary neoplasm and accounts for approximately 3% of all malignancies worldwide with global incidence rates increasing 2–3% per year [1–2]. The clear cell renal cell carcinoma (ccRCC) is the main subtype of RCC and account for approximately 75% of all renal tumors [3]. Surgical management remains the most effective therapeutic alternative for ccRCC. However, there was still about one third patients who develop metastases subsequent to surgery [3]. The prognosis for patients with metastatic RCC is extremely poor largely because of its strong resistance to radiotherapy and chemotherapy and lack of effective therapeutic [4]. Although previous researches have revealed many genetic and epigenetic changes correlated with RCC genesis, there still lack of curative therapy for metastatic RCC and precise mechanism of RCC progression remains poorly understood [5–6]. Therefore, to identify a new reliable and sensible tumor marker for prognostic prediction is crucial for the patients with RCC.

Recent evidence increasingly points to the important role of long non-coding RNA (lncRNAs), the largest transcript class in human genome, which may play an important role in many cellular processes and multiple diseases including cancers [7–9]. Previous researches also reported the expression profile of lncRNA transcript in ccRCC tissues and indicated that there are many dysregulated lncRNAs which may allowed accurate identification of ccRCC tumor tissue [10, 11]. For example, CADM1-AS1 and NBAT-1, may correlated with the progression and worse survival in patients with ccRCC [4, 12]. However, lncRNAs remain poorly characterized in ccRCC.

In this study, through bioinformatics analysis, we performed an integrative analysis of two microarray datasets aimed to identify novel biomarkers of ccRCC. Selected DEGs had been examined in clinical samples by qRT-PCR. We found two high correlated DEGs, peejar and GFOD1 were associated with high ccRCC tumor stage, high ccRCC tumor grade and poor prognosis. Our findings may provide new prognostic biomarkers for patients with ccRCC.

## RESULTS

### Microarray datasets characteristics

ccRCC microarray datasets obtained from ArrayExpress database were included in this study. The E-GEOD-22541 dataset contained 24 primary ccRCC samples, including 8 samples with disease-free survival (DFS) less than or equal to 6 months represented synchronous metastases, 9 samples with DFS greater than or equal to 45 months represented metachronous metastases, and 7 samples without detectable distant metastases after at least 99 months follow up [13]. The E-MTAB-1050 dataset contained gene expression data from 13 primary tumors of ccRCC, and 25 corresponding mice xenograft ccRCC tumors [14]. The E-GEOD-53757 dataset contained 72 paired primary ccRCC samples and normal kidney tissue from the same patient [15]. Detailed description of sample characteristics and clinical variables are provided in the original report [15].

### The gene expression of GFOD1 and peejar is a prognostic marker for ccRCC

Using the Significance Analysis of Microarrays (SAM) method, we identified 998 genes that were differentially expressed between 8 synchronous metastases patients and 16 metachronous metastases patients or patients without detectable metastases disease from E-GEOD-22541 dataset, with standard fold change superior to 1.6 (Supplementary Dataset S1). GO analysis results were shown in Supplementary File S5. Among those DEGs, 138 genes were corresponding to non-coding RNAs (ncRNAs), as known by align each probe sequence to NOCODE (www.noncode.org), LNCipedia (www.lncipedia.ord) [16] and AceView database (http://www.ncbi.nlm.nih.gov/ieb/research/acembly/) [17], using Biostrings bioconductor package (Supplementary Dataset S2). The gene expression correlation between 138 probeset corresponding to ncRNAs and remaining 850 probesets mainly corresponding to coding RNAs were calculated (Supplementary Dataset S3). There are 64 pairs of probesets, corresponding to non-coding or coding respectively, exhibited correlation coefficiency superior to 0.9 (Supplementary Dataset S4).

**Figure 1:**
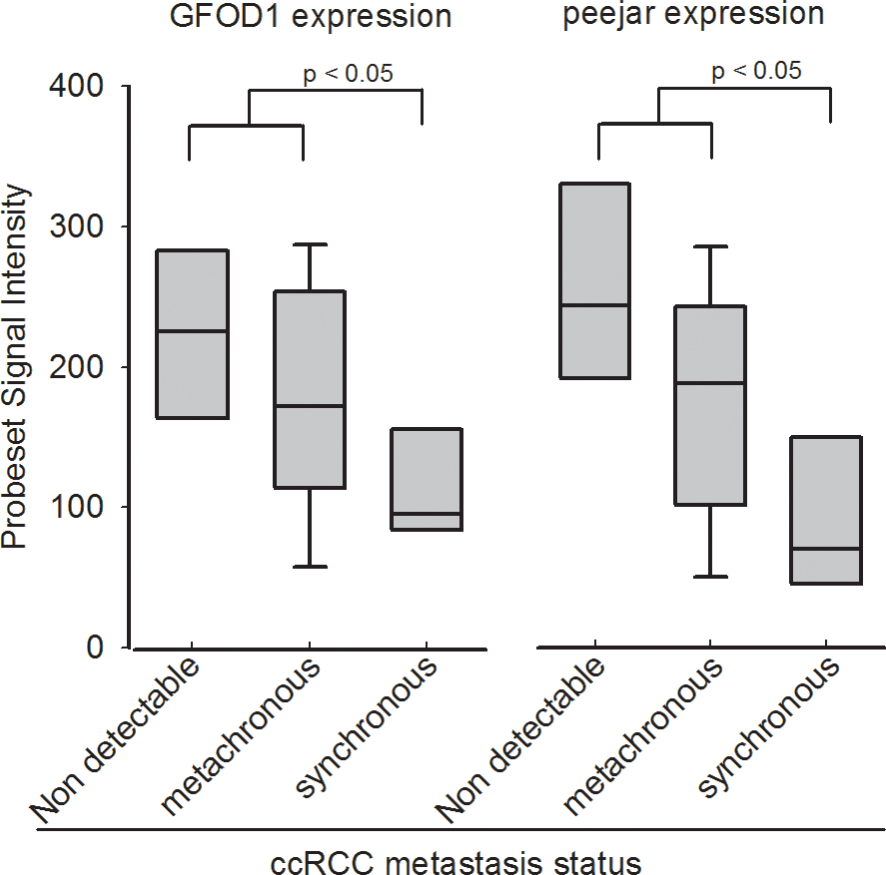
Probeset signal intensity of 219821_s_at (GFOD1) and 230179_at (peejar) from Dataset E-GEOD-22541 Patients with non-detectable metastasis after at least 99 months follow-up shown highest GFOD1 and peejar expression in tumor samples. Patients with synchronous metastasis disease shown lowest GFOD1 and peejar expression in tumor samples, and significant difference compared to patients with non-detectable or metachronous metastasis disease.

DEGs were identified from dataset E-MTAB-1050, using SAM method at false discovery rate = 0.05, and standard fold change cut off = 1.6. GO analysis and KEGG pathway analysis results were shown in Supplementary Files S6 and S7. After overlapping the 64 pair of probesets and the 2910 DEGs (Supplementary Dataset S5), finally 4 pair of genes were selected for next step. There are two DEGs, 219821_s_at and 230179_at, with Pearson correlation coefficiency reaching 0.939 between their signal intensity. They are corresponding to GFOD1 and peejar respectively, after align probe sequences of those two probesets to Aceview database [17] (Supplementary File S1). Gene GFOD1 maps on chromosome 6, from 13487893 to 13363496, on the reverse strand, while gene peejar maps to chromosome 6, from 13363310 to 13358058, on the reverse strand too (NCBI 37, August 2010). There were only 186 bp distance between those two genes on their chromosome, which indicated that GFOD1 gene and peejar gene maybe transcriptional coupling neighboring genes [18]. Their probesets signal intensity had been presented in Figure 1.

### The gene expression of GFOD1 and peejar decreased with ccRCC tumor stage progression

**Figure 2:**
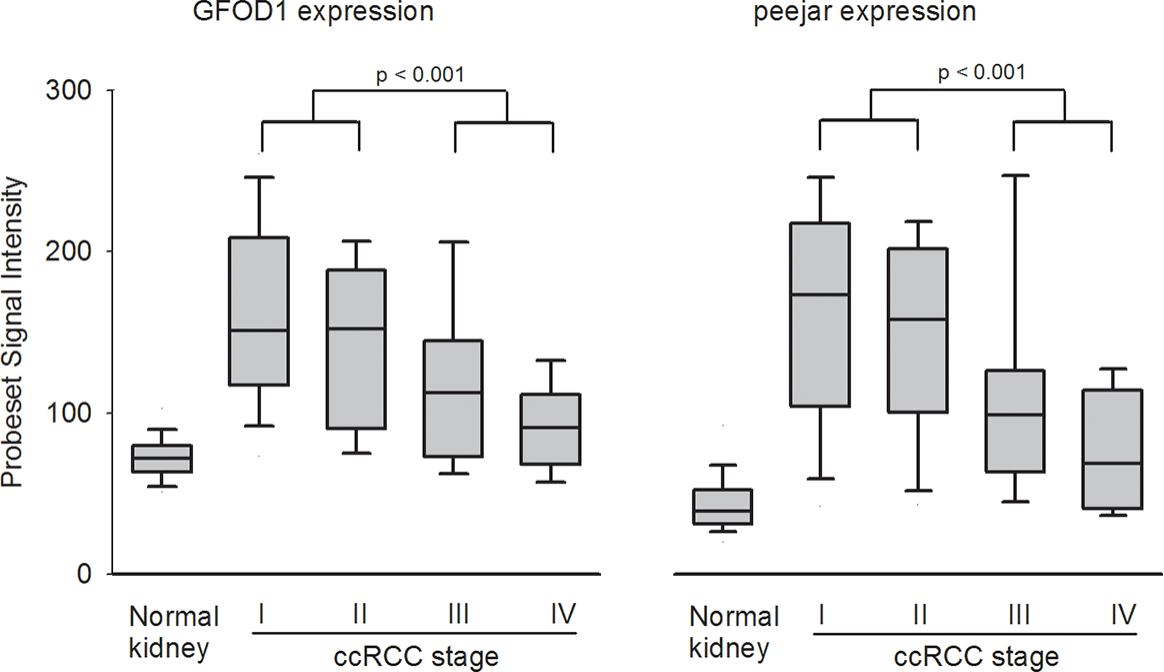
Probeset signal intensity of 219821_s_at (GFOD1) and 230179_at (peejar) from Dataset E-GEOD-53757 Both probesets shown lowest gene expression from 72 normal kidney samples, with highest average gene expression in 24 ccRCC Stage I tumor samples, and gradually decreasing in 19 stage II tumor samples, 14 stage II tumor samples and 15 stage IV samples. There were significant difference between gene expression from patients' samples with early stage and late stage diseases.

The signal intensity of probesets 219821_s_at and 230179_at were extracted from microarray dataset E-GEOD-53757. Both genes exhibited lower expression in normal kidney tissue samples, while significantly elevated expression in stage I ccRCC tumor samples. During disease progression, the gene expression was gradually decreased. There were significant difference between gene expression in early stage (stage I and II) tumor tissue samples and late stage (stage III and IV) tumor tissue samples, which indirectly confirmed the observation that decrease of their gene expression linked to patient's disease progression and poor prognosis (Figure 2).

### The gene expression of GFOD1 and peejar decreased with ccRCC tumor grade progression

**Figure 3:**
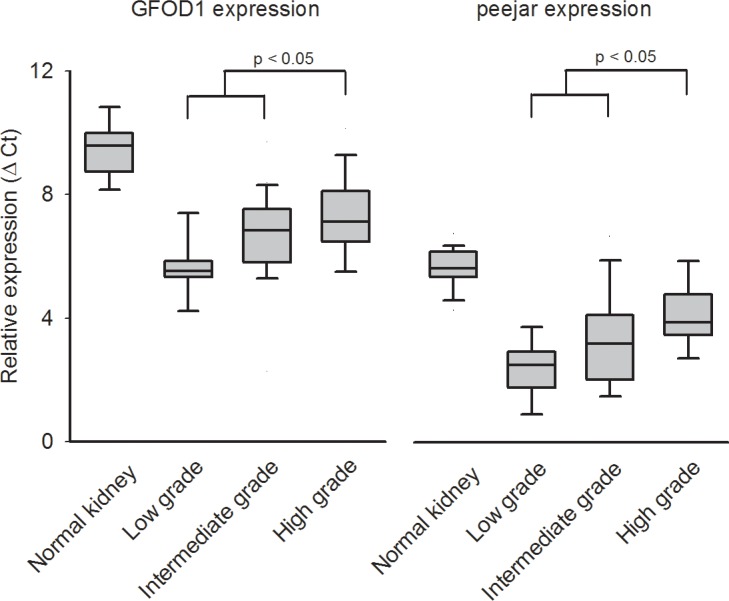
The relative expression (Ct value) from qRT-PCR experiment Higher Ct value indicated lower gene expression. Both GFOD1 and peejar exhibited highest Ct value in normal kidney tissue samples, and lowest Ct value in ccRCC patients' tumor samples with low grade disease. There were significant difference between expression levels from patients' samples with low to intermediate grade and high grade diseases.

We further evaluated the gene expression of GFOD1 and peejar by qRT-PCR in an independent sample of 50 CCRCC tumor samples and 36 non-tumor kidney tissues. After reference genes normalization, the relative expression intensity (Ct) was highest in normal kidney tissue samples, and lowest in tumor samples with low grade G1. There were significant difference between gene expression in low or intermediate grade (G1 and G2) tumor tissue samples and high grade (G3 and G4) tumor tissue samples, which indirectly confirmed the observation that decrease of their gene expression linked to patient's disease progression and poor prognosis (Figure 3).

## DISCUSSION

lncRNAs are a class of RNA molecules arbitrarily defined as being longer than 200 nucleotides and it was initially thought to be a transcriptional noise without protein coding potential [7]. However, lncRNAs have gained massive attention for their crucial roles in gene regulation in recent years [4]. More and more evidence showed lncRNAs may regulate protein coding genes expression at both transcriptional and post-transcriptional level and they were also proved to contribute in tumor development and can be used as biomarkers and prognosis factors [19–20].

Recently, roles of lncRNAs in genitourinary carcinomas have attracted much interest from urological researchers and are becoming a hot spot in renal cancer research [4, 12, 21]. Yao et al. reported lncRNA CADM1-AS1, which is located in the antisense direction of a coding exon of tumor suppressor gene CADM1, was decreased in tumor tissues of ccRCC patients and it may regulate CADM1 expression on proliferation, apoptosis and migration via the expression pattern of “CADM1-AS1/CADM1 mRNA gene pairs” *in vitro* [12]. However, the few examples described above are just a tip of the iceberg as the field of lncRNAs is currently evolving and the work of annotation and characterization of lncRNAs in ccRCC is ongoing [22].

In this study, we retrieve public available ArrayExpress database to identify DEGs which may link to different disease-free survivals. The bioinformatics analysis showed that peejar and GFOD1 expression were significantly elevated in ccRCC tissues, but gradually decreased during cancer progression. We further checked these findings in 50 ccRCC and 36 normal kidney tissues and found the expression of peejar and GFOD1 was significantly higher in low to intermediate grade tumors compared to high-grade tumors, which indirectly confirmed the observation that decrease of their gene expression linked to patient's tumor progression.

However, our study has several limitations. The study population was small and the number of patients with T4 or M1 disease was limited, and it is difficult to compare the qRT-PCR results from patients' samples with early stage disease and late stage disease. The lncRNAs candidates identified here may not represent the complete lncRNA populations underlying ccRCC biological behavior. Although our study revealed the expression of patterns and dysregulation of peejar and GFOD1, their function still remains unknown. peejar and GFOD1 was identified after overlapping two lists of differentially expressed genes. Despite some cancer cells might also expressing GFOD1 and peejar, we are expecting that those GFOD1-expressing tumor infiltrated immune cells, like NK cells, Eosinophil cells and Macrophage cells might contributed to the prognosis (Supplementary Files S8 and S9). Future immunohistochemistry or flow cytometry studies on those immune cells will be focused on checking whether their the quantity difference or GFOD1-expressing level difference could predict the clinical outcome.

In conclusion, our study demonstrated for the first time that lncRNA peejar and mRNA GFOD1 expression was significantly increased in ccRCC and decreased during tumor progression. Our findings indicate the potential roles of lncRNA in ccRCC, and provide useful information for discovery of new biomarkers and therapeutic targets.

## MATERIALS AND METHODS

### Public microarray datasets

ccRCC microarray datasets and corresponding clinical data were retrieved from public available ArrayExpress database [23]. Gene expression profiling was performed using GeneChip human genome U133 Plus 2.0 array (HG-U133Plus2) from Affymetrix (Santa Clara, CA). This array offers 54.000 probe sets for screening 38,500 human genes. Microarray data preprocessing was conducted using the R software and packages from the Bioconductor project [24]. Raw data were collected from CEL files and preprocessed with the Robust Multi-chip Average (RMA) algorithm for background correction, quantile normalization, and median polish summarization [25]. After RMA preprocessing, and nonparametric batch adjustments using ComBat [26], a set of probe ID-centric gene expression values were available for downstream analysis. Differentially expressed genes (DEGs) between any two groups were determined using Significance Analysis of Microarrays (SAM) method with a false discovery rate (FDR) below 0.05, and permutation of 1000. MAplot and histogram were used for analysis processing quality control and PCA analysis to evaluate the quality of processed data (Supplementary File S2).

### Patients and samples

We included 50 ccRCC tumor samples and 36 non-tumor kidney tissues for qRT-PCR analyses. The tumors were staged according to the TNM system developed by the American Joint Committee on Cancer and the International Union against Cancer [27] and graded according to Fuhrman's nuclear grading system [28]. Out of the 50 tumors, 10 in low grade G1, 23 in intermediate grade G2, 15 in high grade G3, and 2 in high grade G4, while 35 in stage I, 9 in stage II, and 6 in stage III. The written informed consent was obtained from all participants. The study was approved by the Ethics Committee of Ren Ji hospital, China. The detailed clinical information is provided in Supplementary File S3.

### RNA extraction and qRT-PCR experiments

Frozen tissues were grossly dissected into TRIZOL for RNA extraction following standard protocols using dry ice pre cold pestle and motor. Using an Anakytik Jena scandrop spectrophotometer, the ratio of the absorbance of each RNA at 260 and 280 nm (A260:A280) was measured as an indicator of RNA purity. The mRNA integrity was assessed by the absorption curve, which showed two clear rRNA bands of 28S and 18S. One microgram of total RNA was used for cDNA synthesis with QuantiTect reverse transcription kit (Qiagen). qRT-PCR was performed on an ABI 7900HT instrument using QuantiFast SYBR green PCR kit (Qiagen). A combination of 4 genes, HMBS, PPIA, ATP5J and TBP, being stably expressed in tissues from ccRCC, has been used as reference genes for gene expression internal control [29]. Primer sequences of targets are provided in Supplementary File S4. Each amplification was run in duplicate. The cycle number of each target was normalized against the geometric mean of the cycle numbers from 4 reference genes. The calculation of delta Ct value was performed as follows: Ct(target) = Ct (target) − GM{Ct(HMBS), Ct(PPIA), Ct(ATP5J), Ct(TBP)}.

### Statistical analysis

The DEGs expression differences between the tumor and normal kidney samples were analyzed using Student's *t*-test with SPSS software (version 17.0, SPSS). A two-sided *p* value less than 0.05 was considered to be statistically significant.

## SUPPLEMENTARY MATERIALS FIGURES AND TABLES












