# Supplementary material for: GFOD1 and peejar are promising markers for clear-cell renal cell carcinoma disease progression
Source: Oncotarget. 2016 May 15;7(25):38004–9. doi: 10.18632/oncotarget.9379 (PMC5122367; doi:10.18632/oncotarget.9379)
Supplement: Supplementary file 1 [file oncotarget-07-38004-s001.pdf]

GFOD1 and peejar are promising markers for clear-cell renal cell carcinoma disease progression

Supplementary Materials

Supplementary File S1: GFOD1 and peejar selection flow chart.

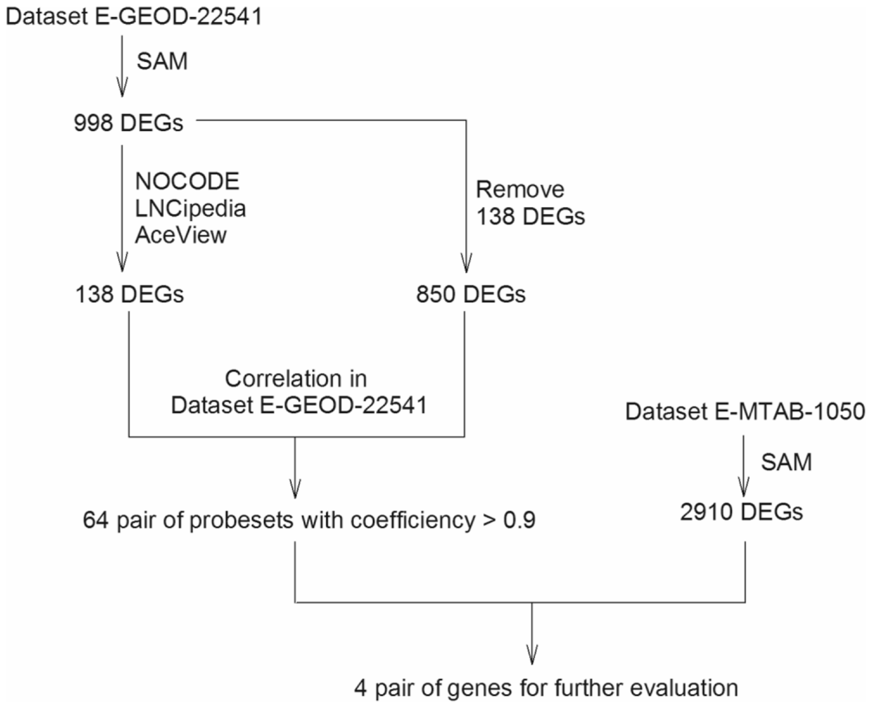

List of 4 pair of genes

	Annotation	Chromosome		Annotation	Chromosome
201090_x_at; 211058_x_at; 213646_x_at	TUBA1B	Ch12	209251_x_at; 211750_x_at; 212639_x_at	TUBA1C	Ch12
219821_s_at	GFOD1	Ch6	230179_at	peejar	Ch6
231947_at	MYCT1	Ch6	213891_s_at	TCF4	Ch18
205326_at	RAMP3	Ch7	225809_at	PARM1	Ch4

Differential expression genes (DEGs) were identified from dataset E-GEOD-22541, using statistical analysis of microarrays (SAM method) at false discovery rate = 0.05, and standard fold change cutoff = 1.6. We aligned each probe sequence from totally 998 DEGs with three databases, including NOCODE, an integrated database for non-code RNA (www.noncode.org); LNCipedia, a resource for lncRNA transcripts (www.lncipedia.ord); and AceView, a comprehensive sequence representation of all public RNA sequences including both coding and non-coding RNA (http://www.ncbi.nlm.nih.gov/ieb/research/acembly/). Totally 138 DEGs were supposed to mainly represented non-coding RNA, and residue 850 DEGs were mainly represented to coding RNA. There are 64 pairs of probesets, corresponding to non-coding or coding respectively, exhibited correlation coefficiency superior to 0.9. Differential expression genes (DEGs) were identified from dataset E-MTAB-1050, using statistical analysis of microarrays (SAM method) at false discovery rate = 0.05, and standard fold change cutoff = 1.6. After overlapping the 64 pair of probesets and the 2910 DEGs, finally 4 pair of genes were selected for next step. GFOD1 and peejar were selected for further study, since they were located in the same chromosome, and there were only 186 bp distance between those two genes on their chromosome. Potentially GFOD1 gene and peejar gene were transcriptional coupling neighboring genes.

Supplementary File S2: Quality control microarray datasets. MAplot and histogram were used for analysis processing quality control. Generally speaking, the arrays downloaded from dataset E-GEOD-22541 and dataset E-GEOD-1050 were performed with high quality and the RMA algorithm is ok for data analysis. We performed MAplot comparison before and after normalization. The demonstrative comparison figures were presented below

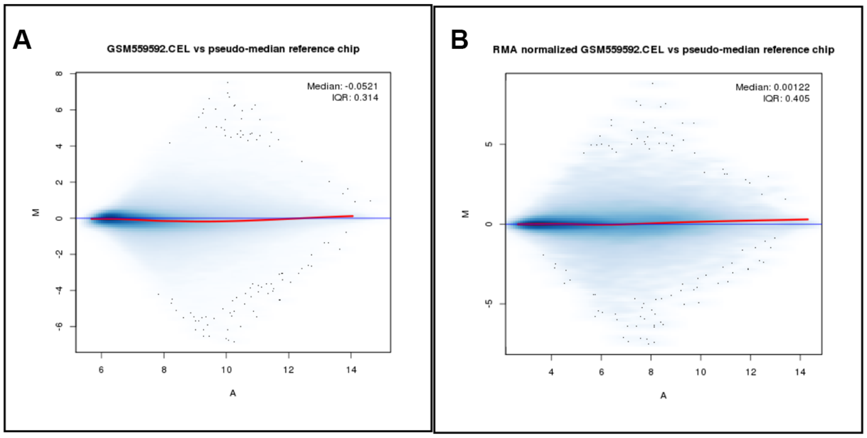

MAplot for array number GSM559592.CEL, before normalization (**A**) and after normalization (**B**).

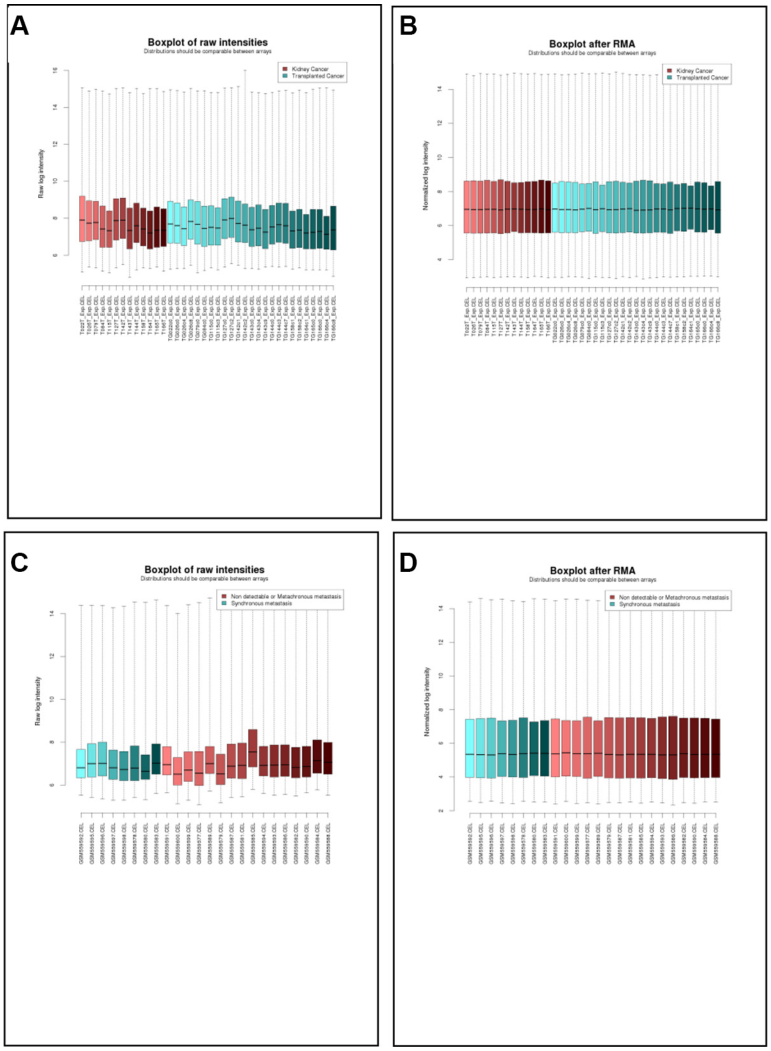

Histogram for dataset E-GEOD-1050 (A and B) and dataset E-GEOD-22541 (C and D), before normalization (A and C) and after normalization (B and D), to indicate the efficiency of RMA normalization.

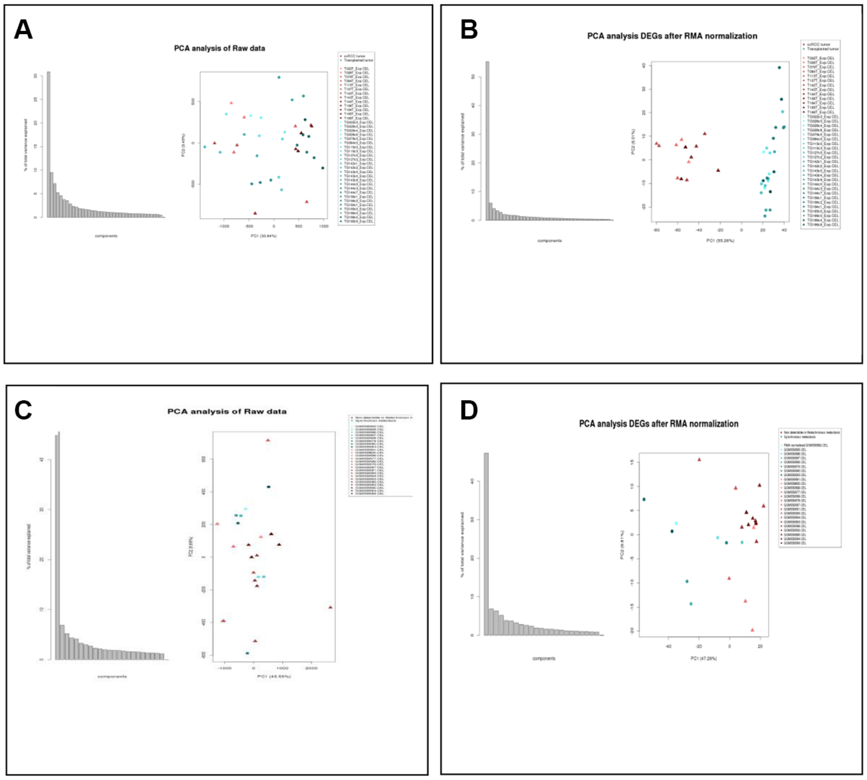

PCA analysis for raw data and selected DEGs, for dataset E-GEOD-1050 (A and B) and E-GEOD-22541 (C and D), using whole data (A and C) or selected differentially expressing genes (B and D).

Supplementary File S3: Clinical information of the ccRCC patients

ccRCC Patients	Gender	Age (year)	Furhman Grade	T Stage (TNM)	Clinical Stage
T01	Male	68	IV	1B	1
T02	Male	60	III	1B	1
T03	Male	78	III	1B	1
T04	Female	34	III	1A	1
T05	Female	70	III	1A	1
T06	Male	53	III	1A	1
T07	Male	60	III	1A	1
T08	Male	61	III	3A	3
T09	Female	30	III	2A	2
T10	Male	48	III	1A	1
T11	Male	67	III	3B	3
T12	Male	52	III	1A	1
T13	Male	59	III	1B	1
T14	Male	88	III	1A	1
T15	Male	57	III	2A	2
T16	Male	62	II	1B	1
T17	Male	61	II	3A	3
T18	Female	71	II	1B	1
T19	Female	45	II	1B	1

T20	Male	58	II	1B	1
T21	Male	63	II	2A	2
T22	Female	57	II	2A	2
T23	Female	52	II	2A	2
T24	Male	47	II	1B	1
T25	Male	59	II	2B	2
T26	Male	69	II	1B	1
T27	Male	65	II	1B	1
T28	Female	84	II	1B	1
T29	Male	57	II	2A	2
T30	Male	56	I	3A	3
T31	Male	58	I	1B	1
T32	Male	36	I	1A	1
T33	Female	45	I	1B	1
T34	Male	40	I	1B	1
T35	Male	41	I	1B	1
T36	Male	47	I	1B	1
T37	Male	58	I	1A	1
T38	Female	56	I	1A	1
T39	Female	52	I	1A	1
T40	Female	66	II	1B	1
T41	Male	65	II	1B	1
T42	Female	61	IV	2B	3
T43	Male	59	II	2a	3
T44	Male	74	III	2A	2
T45	Male	61	II	2A	2
T46	Female	79	II	1A	1
T47	Male	61	II	1A	1
T48	Male	48	II	1A	1
T49	Female	63	II	1A	1
T50	Female	56	II	1A	1

Supplementary File S4: Primer sequences for the targets and reference genes

	Primer 1	Primer 2
peejar	ACTTACTTGTGATTCTTGGTCTAA	GCAAAGGAATGGGTCAGAT
GFOD1	GCCTTCAGTTCCAATCAG	ATAATGTTCCTTGTTGTCCTT
HMBS	ATACAGCTATGAAGGATGG	AGTGATGCCTACCAACTG
PPIA	GGCATGAATATTGTGGAG	GGAATGATCTGGTGGTTA
ATP5J	CATTGGTGTTACAGCAGTG	CAACAGGTCCTCCAGATG
TBP	CAGTGAATCTTGGTTGTAA	TGGCTCTCTTATCCTCAT

Supplementary File S5: Top 15 enriched GO terms of DEGs identified from dataset E-GEOD-22541

GO ID	GO annotation	*p*-value
GO:0008380	RNA splicing	1.10E–09
GO:0006397	mRNA processing	3.86E–08
GO:0016071	mRNA metabolic process	7.57E–08
GO:0006396	RNA processing	1.78E–07
GO:0044260	cellular macromolecule metabolic process	4.84E–07
GO:0034641	cellular nitrogen compound metabolic process	4.41E–06
GO:0000377	RNA splicing, via transesterification reactions with bulged adenosine as nucleophile	5.38E–06
GO:0000398	nuclear mRNA splicing, via spliceosome	5.38E–06
GO:0000375	RNA splicing, via transesterification reactions	5.38E–06
GO:0006139	nucleobase, nucleoside, nucleotide and nucleic acid metabolic process	6.66E–06
GO:0006807	nitrogen compound metabolic process	7.32E–06
GO:0016070	RNA metabolic process	1.41E–05
GO:0043170	macromolecule metabolic process	4.89E–05
GO:0000280	nuclear division	5.18E–05
GO:0007067	Mitosis	5.18E–05

Supplementary File S6: Top 15 enriched GO terms of DEGs identified from dataset E-MTAB-1050

GO ID	GO annotation	*p*-value
GO:0002376	immune system process	1.61E–44
GO:0006955	immune response	6.85E–39
GO:0006952	defense response	1.93E–20
GO:0002682	regulation of immune system process	6.07E–18
GO:0050896	response to stimulus	1.57E–17
GO:0002684	positive regulation of immune system process	3.46E–15
GO:0001775	cell activation	7.75E–15
GO:0007165	signal transduction	1.97E–14
GO:0045321	leukocyte activation	6.08E–14
GO:0050776	regulation of immune response	9.56E–14
GO:0007166	cell surface receptor linked signal transduction	1.30E–13
GO:0006954	inflammatory response	1.41E–13
GO:0009611	response to wounding	9.03E–13
GO:0046649	lymphocyte activation	1.78E–12
GO:0050865	regulation of cell activation	2.05E–12

Supplementary File S7: KEGG pathway analysis of DEGs identified from dataset E-MTAB-1050.

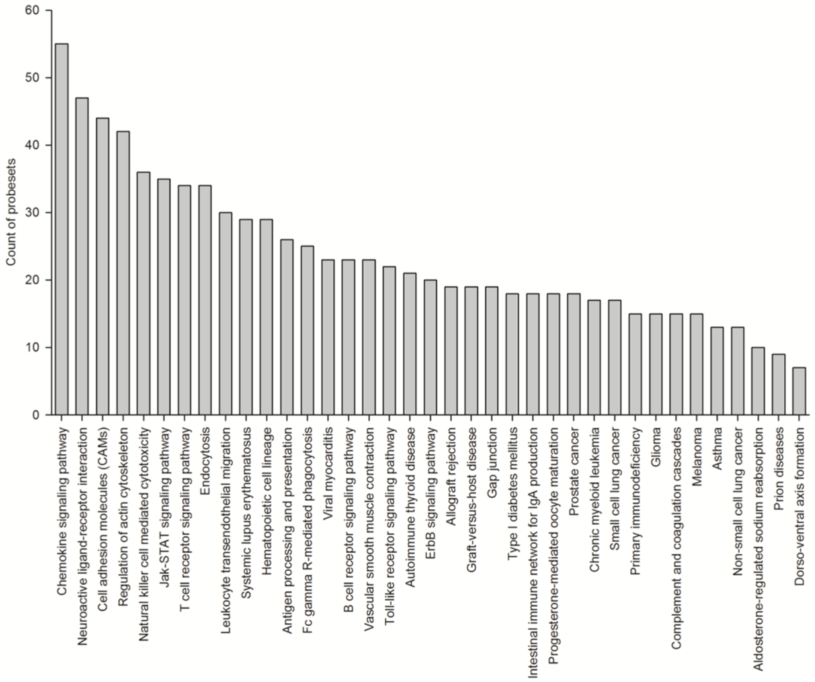

The data exhibited are significantly enriched with immune cell mediated signal pathways.

Supplementary File S8: Tumor infiltrated immune cells might contributed to both GFOD1 and peejar expression.

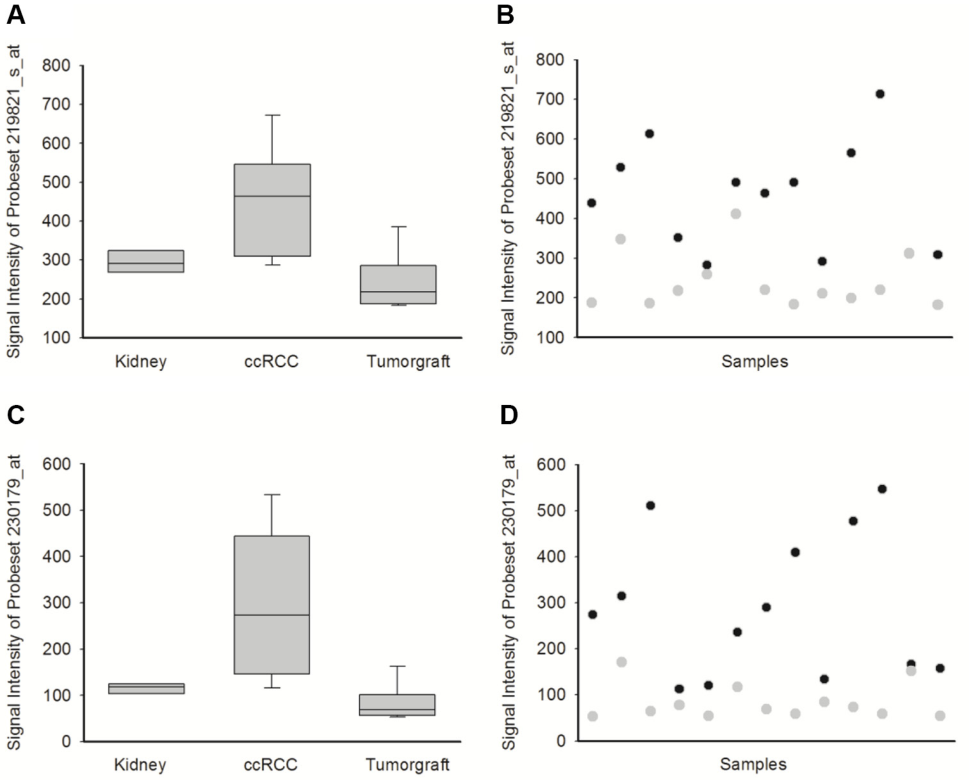

Probeset signal intensity extracted from dataset E-MTAB-1050. Both probeset 219821_s_at (GFOD1) and probeset 230179_at (peejar) shown some expression in normal kidney samples, and such expression were significantly elevated in ccRCC tumor samples, but reduced to significantly lower level expression in tumor Xenograft samples (**A** and **C**). The paired comparison between ccRCC tumor and xenograft samples were shown in **B** (GFOD1) and **D** (peejar), exhibited distinct difference in most samples.

Supplementary File S9: Both Eosinophils and NK cells were positive for GFOD1 and peejar in blood samples.

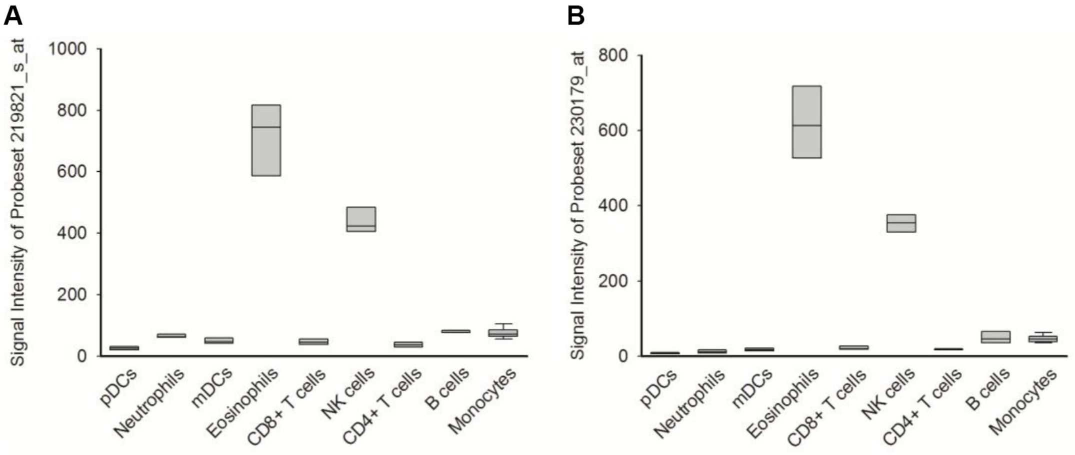

Probeset signal intensity extracted from dataset E-GEOD-28490. Both probeset 219821_s_at (GFOD1) and probeset 230179_at (peejar) shown high expression level in Eosinophils and NK cells, but relatively weak expression in other cells, like pDCs, neutrophils, mDCs, CD8+ T cell, CD4+ T cells, B cells and monocytes. Tumor micro environment also enriched with macrophages, and a sub group of microphage might be also positive for GFOD1 and peejar.

Supplementary Dataset S1: Differentially expressed genes from E-GEOD-22541 dataset were identified using the significance analysis of microarrays method. See Supplementary_Dataset_S1

Supplementary Dataset S2: 138 DEGs was selected after compare 998 DEGs with three different non coding RNA databases. See Supplementary_Dataset_S2

Supplementary Dataset S3: The gene expression correlation between 138 DEGs (non-coding RNAs) and 850 DEGs (coding RNAs). See Supplementary_Dataset_S3

Supplementary Dataset S4: 64 pair of probesets with correlation coefficiency > 0.9. See Supplementary_Dataset_S4

Supplementary Dataset S5: Differentially expressed genes from E-MTAB-1050 dataset were identified using the significance analysis of microarrays method. See Supplementary_Dataset_S5
